# An updated review on systemic glucocorticoids in the prevention and treatment of bronchopulmonary dysplasia

**DOI:** 10.1002/pdi3.6

**Published:** 2023-06-12

**Authors:** Xinyi Wang, Yuan Shi

**Affiliations:** ^1^ Department of Neonatology Children's Hospital of Chongqing Medical University Chongqing China; ^2^ Ministry of Education Key Laboratory of Child Development and Disorders Chongqing China; ^3^ National Clinical Research Center for Child Health and Disorders Chongqing China; ^4^ China International Science and Technology Cooperation Base of Child Development and Critical Disorders Chongqing China; ^5^ Chongqing Key Laboratory of Pediatrics Chongqing China

**Keywords:** BPD, bronchopulmonary dysplasia, preterm, systemic glucocorticoids

## Abstract

Bronchopulmonary dysplasia (BPD) is a severe chronic pulmonary disease affecting premature infants and their families. The main pathogenesis of BPD is the abnormal inflammatory response in immature lungs, resulting in the distortion of lung development and influencing the formation of alveoli and vascular structures. Based on anti‐inflammatory mechanisms, glucocorticoids may reduce the incidence and mortality of BPD in premature infants, but different types and administrations of glucocorticoids may lead to various short‐term or long‐term outcomes, especially the most concerning neurodevelopmental abnormalities. Dexamethasone is currently recognized as a first‐choice drug for BPD, but the different administration methods may affect the efficacy and safety of the regimen. Hydrocortisone is currently considered to minimize both short‐term and long‐term adverse outcomes, but its effectiveness needs to be confirmed by further trials. Numerous alternative strategies have been proposed to reduce adverse reactions, such as moderate early dosing, lower cumulative doses, and individualized dosing, but the high‐quality evidence is still not enough. This review is trying to summarize some new developments in mechanisms of action, adverse effects, and other treatment regimens of systemic glucocorticoids for the prevention and treatment of BPD in the recent few years.

## Graphical abstract

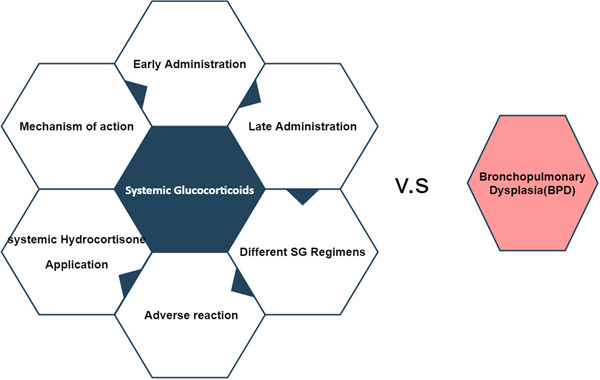

## 1 INTRODUCTION

The incidence of bronchopulmonary dysplasia (BPD) is still increasing each year,^1^ which severely affects premature infants, especially those born at less than 28 weeks of gestation. Northway et al. were the first to describe BPD. Initially, “BPD” was utilized to describe the pulmonary manifestations that emerge from the superimposed healing of mechanical ventilation (MV)‐induced damage and respiratory distress syndrome.^2^ With the successful application of exogenous surfactant therapy, the “old BPD” mainly characterized by airway injury, inflammation, and parenchymal fibrosis is no longer applicable and replaced by the “new BPD”, which is characterized by reduced fibrosis, airway injury, and uniform alveolar expansion during simplification.^3^ Therefore, BPD has been redefined as lung injury in preterm infants who require supplemental oxygen for more than 28 days after birth. Based on the amount of supplemental oxygen required at 36 weeks postmenstrual age (GA < 32 weeks) or after 28 days (GA ≥ 32 weeks), BPD is categorized as mild, moderate, and severe.^1^, ^2^

Neonatologists worldwide have made many efforts to prevent it from causing severe harm to patients and their families, but the currently available treatment for BPD is still limited. The inflammatory response is generally considered to be the primary pathogenesis of BPD,^4^ and thus, glucocorticoids are often used for the prevention and treatment of BPD^5^ with their potent anti‐inflammatory effects. The current clinical use of glucocorticoids for BPD mainly includes inhaled and systemic use, prenatal and postnatal administration, and postnatal glucocorticoids have been certified and proven as the only pharmacologic interventions.^6^ Dexamethasone and hydrocortisone are the most commonly used postnatal systemic corticosteroids. Numerous previous trials have been conducted to assess the best administration time and dose of dexamethasone and hydrocortisone in the management of BPD. Unfortunately, these findings have demonstrated significant variations. In addition, systemic glucocorticoids (SG) have been reported to be associated with some short‐term and long‐term adverse outcomes while lowering the risk of BPD, such as neurodevelopmental problems.^7^, ^8^ Current research is focusing on whether postnatal corticosteroids should be used and how to be used effectively so that the benefits can outweigh the risks. Therefore, this review aims to summarize the existing literature and try to provide some further evidence‐based suggestions for SG management in the prevention and treatment of BPD.

## 2 PREVALENCE AND CLINICAL FEATURES OF BPD

BPD is one of the most common sequelae of prematurity and has significant prognostic implications. Birth weight and gestational age are the two main risk factors for BPD with the incidence rising with falling birth weight and gestational age. Sepsis, intrauterine growth restriction, oxygen delivery, and prolonged mechanical breathing are additional significant risk factors.^9^, ^10^, ^11^ BPD affects at least a quarter of infants with birth weights of less than 1500 g. In the United States, it affects 10,000–18,000 infants per year, including approximately 50% of those with a birth weight of less than 1000 g.^12^

In the following decades, the introduction of prenatal glucocorticoids, milder ventilation strategies, and surfactant therapy altered the pathophysiology of BPD, replacing the original concept with a “new BPD” characterized by reduced fibrosis, airway damage, and homogeneous alveolar expansion during simplification.^13^ The “new BPD” primarily affects infants born at less than 1500 g and 32 weeks of gestation and is now rare in infants born at more than 1500 g and 32 weeks of gestation.

Since 2000, some reports suggest that BPD incidence has begun to decline, but most studies suggest that BPD rates have remained stable or even increased over the past 2–30 years likely due to improved survival rates for at‐risk infants with advances in modern medicine. Incidence rates of BPD for babies born 501–1500 g between 1997 and 2018 have been reported at 23.2%–32.6%.^14^ There was no clinically relevant decline during this period.

Although several individual risk factors have been identified, BPD may result from a complex interaction between immature lungs and multiple perinatal and postnatal exposures. Patients with BPD are at risk for chronic sequelae, including long‐term lung deficits, neurodevelopmental disorders, and late neonatal mortality. There is evidence that many of these sequelae persist into adulthood even in children born in the “new BPD” era.^13^

To reduce the risk of BPD, the main clinical approaches are prevention of preterm birth, use of noninvasive ventilatory measures, administration of surfactant, caffeine, vitamin A, and glucocorticoids, and recent emerging treatment strategies, such as inhaled nitric oxide and cell therapy, but their efficacy and safety need to be confirmed by further clinical trials.^15^

## 3 MECHANISM OF ACTION OF GLUCOCORTICOIDS

Infected lung damage, oxygen toxicity, and MV all contribute to the pathogenesis of BPD in premature newborns; the crucial mechanism is that the above events cause an excessive inflammatory response.^16^ The inflammatory response induced in immature lungs is essential in distorting pulmonary development. It affects alveolar, mesenchymal, and vascular structures, ultimately leading to abnormal tissue repair and arrest of pulmonary development, causing newly developed BPD, whose pathological basis is vascular dysregulation and defective alveolar development.^4^

The excessive inflammatory response involves many mechanisms, most studied of which are the breaking of the balance between pro‐inflammatory cytokines and lung development‐promoting factors, the attraction of innate immune cells to the immature lung, breaking of the balance of cytokine signaling networks, phenotypic distortion of lung‐resident mesenchymal stem cells, and the dual feature of inflammatory cytokine signaling pathways.^4^ In the inflammatory response, pro‐inflammatory cytokines are overproduced, including interleukin‐1β (IL‐1ß), interleukin‐6 (IL‐6), interleukin‐8 (IL‐8), and tumor necrosis factor‐α (TNF‐α), granulocyte colony‐stimulating factor (GCSF), macrophage inflammatory proteins (MIP), and monocyte chemotactic proteins (MCP). In contrast, anti‐inflammatory cytokines, such as interleukin‐10, are downregulated.^17^ Meanwhile, the overexpression of pro‐inflammatory mediators attracts alveolar macrophages and neutrophils as well, leading to an innate immune response that induces a shift from an initial M2 anti‐inflammatory state to an M1 inflammatory state, which predominates and releases more pro‐inflammatory cytokines, continuing to enhance the inflammatory process in the lung.^18^, ^19^, ^20^ At the same time, the overactivation of the TGF‐β inhibits lung myofibroblast function and platelet‐derived growth factor (PDGF) receptor α‐mediated vascular endothelial growth factor A (VEGFA) secretion, resulting in the dysregulation of vascular development. Inflammatory cytokines play a double role in lung development and injury. However, they may exacerbate damage of the lungs when overstimulated, resulting in distorted lung development and thus forming the pathological basis of newly emerging BPD.^15^

**FIGURE 1:**
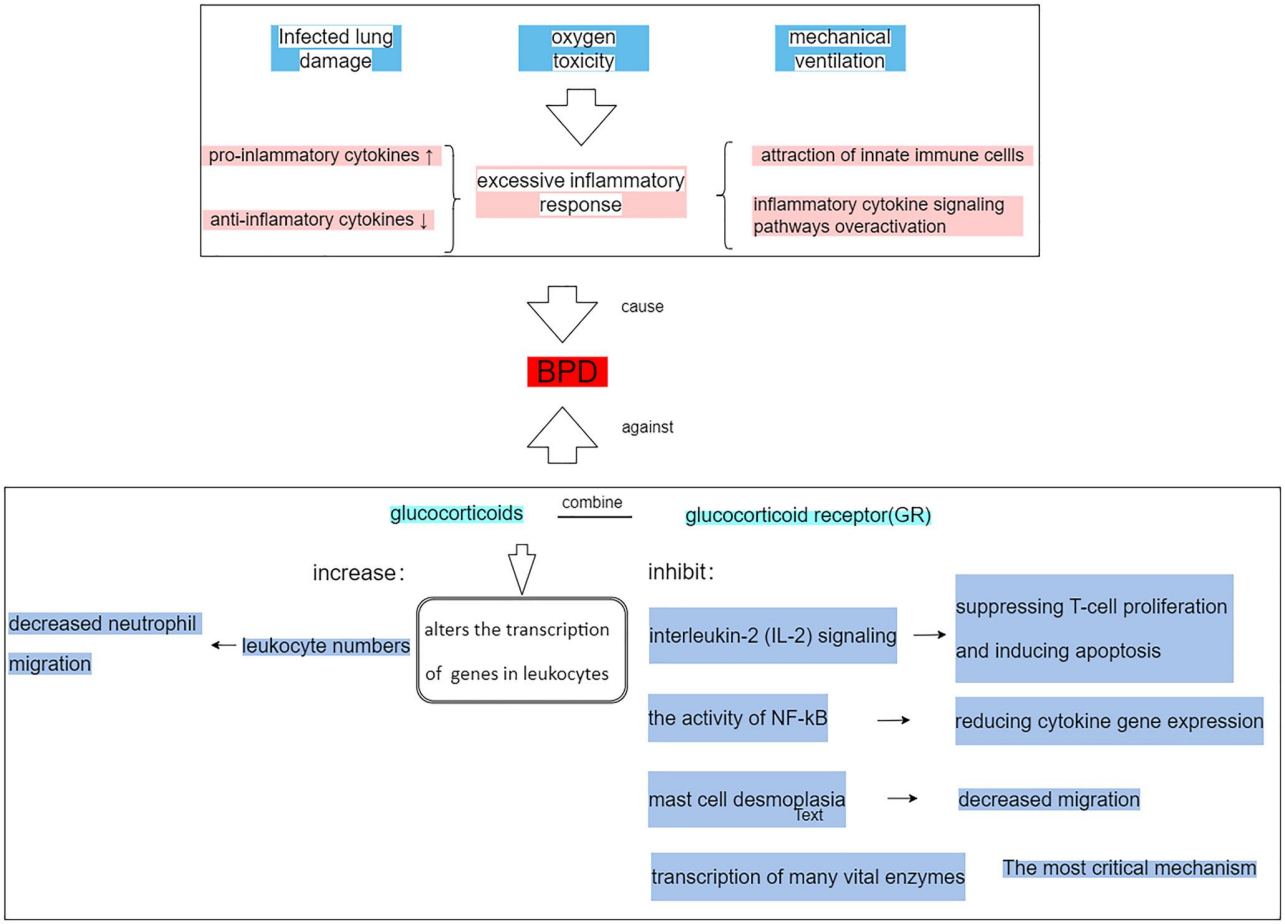
The pathogenesis of BPD and the mode of action of systemic glucocorticoids. BPD, bronchopulmonary dysplasia.

Glucocorticoids exert their anti‐inflammatory roles primarily through the transcriptional effect of the glucocorticoid receptor (GR), which alters the transcription of numerous genes in leukocytes, including their up or downregulation.^5^ Glucocorticoids cause a net increase in leukocyte numbers, resulting in decreased neutrophil migration into tissues; they also inhibit interleukin‐2 (IL‐2) signaling, thereby suppressing T‐cell proliferation and inducing apoptosis; inhibit the activity of NF‐kB, a critical transcriptional regulator of pro‐inflammatory genes, thereby reducing cytokine gene expression; and inhibit mast cell desmoplasia.^21^ The most critical mechanism is glucocorticoid‐inhibiting transcription of many vital enzymes encoding pro‐inflammatory cytokines and chemokines, cell adhesion molecules, and those involved in initiating or maintaining the host inflammatory response.^22^ A more detailed understanding of the pathogenesis of BPD and the mechanism of glucocorticoid action may provide a reference to better utilize the roles of glucocorticoids. Figure 1 briefly summarizes the pathogenesis of BPD and the mode of action of SG.

## 4 APPLICATIONS OF SYSTEMIC GLUCOCORTICOIDS

Glucocorticoids have potent anti‐inflammatory functions and are pivotal in respiratory epithelial cell development and lung maturation. Therefore, these efficient anti‐inflammatory drugs have long been used for BPD. Since the 1980s, we often use SG in managing infants with BPD in clinical practice.^1^ Systemic glucocorticosteroids, which largely use dexamethasone and hydrocortisone, and inhaled glucocorticosteroids, which include beclomethasone, budesonide, fortikasone, fornizotide, and dexamethasone, are the two major classes of glucocorticosteroids most commonly used in clinical settings.^23^

The current clinical protocols for prophylaxis and curing BPD with glucocorticoids are prenatal and postnatal glucocorticoids, including systemic and inhaled glucocorticoids. However, the risk/benefit ratio of their use remains controversial. Antepartum glucocorticoid administration has been proven to promote fetal lung maturation in preterm infants, reducing respiratory distress syndrome and mortality.^24^ However, there are no recent developments regarding their effect on lowering the BPD incidence rate, so they are not highlighted in this article. Postnatal systemic administration (PNS) of glucocorticoids reduces the incidence and severity of BPD. However, it is accompanied by long‐term neurodevelopmental and respiratory consequences, so routine drug administration is not recommended. Glucocorticoids may lead to an increased risk of neurodevelopmental disorders.^24^ Alternative glucocorticoid regimens have been investigated over the last few years, covering the timing and duration of steroid initiation, pulsed or non‐pulsed administration, and cumulative cortisol regimens to maximize the benefits of glucocorticoids and to avoid short‐term and long‐term negative consequences.^7^ Hydrocortisone is a synthetic cortisol analog with glucocorticoid activity, but it exhibits saline corticosteroid activity at supraphysiological doses. While hydrocortisone has nothing to do with long‐term detrimental neurodevelopmental impacts, its early use might reduce the incidence or death of BPD.^25^ There is insufficient evidence to demonstrate the efficacy of hydrocortisone against BPD, and thus further trials are needed.

### 4.1 Early administration of SG

Initially, SG were first applied to facilitate extubation. In the 1990s, when Dexamethasone began to be used, high cumulative doses (i.e., 6.16 mg/kg) were administered early in life (12 h), and a prolonged course (49 days) of Dexamethasone resulted in short‐term adverse effects, such as transient hyperglycemia, hypertension, sepsis, hyperparathyroidism, and growth retardation.^3^ In 2000, Yeh et al. reported a significant decrease in indicators of neurodevelopment, including motor skills and IQ scores, in children receiving dexamethasone therapy early in their life, causing awareness of early dexamethasone application that may have long‐term negative consequences—neurodevelopmental disorders. Therefore, early SG therapy for BPD is not recommended.

In 2021, to verify the effect of dexamethasone and hydrocortisone during the first six days, Doyle et al. elected 32 randomized controlled trials involving 4395 preterm newborns. The main results showed that early SG had little or no overall effect on mortality; on the contrary, early SG increased the risk of cerebral palsy (CP), gastrointestinal perforation, and combined mortality. Besides, strong data showed that dexamethasone alone and early SG had the same effect on the combined outcome of mortality or BPD at 36 weeks. Furthermore, in the Doyle et al. study, early dexamethasone use had negligible influence on the combined outcome or BPD, contrary to the prevailing view that early dexamethasone appears to increase this combined outcome.^26^

While there was substantial evidence that hydrocortisone alone could lower mortality, it had no impact on reducing the total incidence of BPD at postmenstrual age (PMA) 36 weeks (high‐certainty evidence). Contrarily, hydrocortisone had little influence on the combined outcome of death and decreased the combined outcome of mortality or BPD at 36 weeks (high‐certainty evidence).^26^ Briefly, Doyle et al. concluded that early SG is helpful as well as harmful for BPD in preterm children with a high‐risk condition and that administering dexamethasone early was relevant to both advantageous and detrimental effects but did not seem to lower mortality. The benefit of early hydrocortisone use for BPD control has not been clinically proven, while it perhaps will help prevent death without impairing neurodevelopment. Because the evidence is insufficient, further trials are needed on neurodevelopmental disorders and other long‐range impacts.

In 2022, Thangaraj et al. concluded that early systemic steroid use, whether dexamethasone or hydrocortisone, was associated with a lower risk of BPD or mortality (moderate strength of evidence), and strong data indicated that early systemic dexamethasone use was associated with a lower risk of BPD or mortality at PMA 36 weeks PMA, but that use of dexamethasone early was associated with a poor long‐term neurologic prognosis.^6^ Thus, up to now, there remains an international consensus against the application of systemic dexamethasone in the first week of life in neonates.

### 4.2 Late administration of SG

In 2021, Doyle et al. evaluated 21 randomized controlled trials involving 1382 infants, incorporating two randomized controlled trials (435 infants) evaluating hydrocortisone alone. High‐certainty data showed a significant cut‐down in mortality with late SG, and the incidence of BPD also decreased at 36 weeks of PMA (moderate‐certainty data). The same result was observed for the combined outcome of mortality or BPD at 36 weeks of PMA. For another, the high‐certainty data showed that late SG had almost no effect on CP and mortality or the combined outcome. Dexamethasone has been demonstrated in subgroup analysis to reduce the incidence of BPD at PMA 36 weeks, but hydrocortisone has not been shown to have the same impact. Therefore, Doyle et al. concluded that late SG is beneficial for BPD, and late SG reduces mortality or BPD at 36 weeks of PMA without significantly increasing CP.^27^ However, more prognosis data are lacking; thus, late SGs ought to be used with caution in newborns who are unable to be weaned from mechanical breathing, beginning at exceeding 7 days of age and for the shortest possible time and dose.

In 2022, a systematic review and meta‐analysis of 14 SG regimens revealed that the most appropriate regimen of treatment for lowering mortality or the development of BPD at 36 weeks of PMA was moderately early use (8–14 days), moderate cumulative dose (2–4 mg/kg), and short duration (<8 days).^6^ There was no evidence that the evaluated interventions were associated with the risk of neurodevelopmental disorders. Nevertheless, no studies are available to research clinical diversities in the occurrence of remote neurologic adverse events. International advisory bodies currently recommend an accumulated dose of 1–2 mg/kg of dexamethasone for exceeding 7–10 days.^7^ Overall, late SG is thought to be favorable for BPD in preterm newborns, lowering mortality and incidence of BPD at 36 weeks of PMA, although there is little evidence to support its long‐term impact on neurologic events. Therefore, further clinical trials are needed to evaluate the appropriate start date, cumulative dose, and duration of treatment.

### 4.3 Adverse reactions of SG

The first is the most concerning issue of neurological development. Hemasree et al. examine the relationship between postnatal age (PNA) at the first use of systemic steroids at 29 weeks gestation and mortality at corrected age 18–24 months or significant neurodevelopmental disorders (sNDI) at corrected age 18–24 months by analyzing follow‐up data from 6200 babies exposed to systemic steroids for BPD after the first week in a retrospective cohort study.^28^ The results showed no significant correlation between mortality or the composite outcome of sNDI and the PNA of sPNS. Beyond that, a delay in the timing of the initial application of systemic steroids among survivors might have increased the odds of sNDI and motor delay, but confounding factors like birth weight, gestational age, and whether they were affected by exposure to antenatal steroids have not been excluded, so the effects need to be proven in additional clinical trials. Noura et al. analyzed 3662 newborns of whom 901 (24.6%) were diagnosed with BPD. According to Noura et al., PNS was linked to a significant rise in the incidence of dyskinesia in the BPD group at the highest risk despite the fact that this effect persisted regardless of the steroid utilized.^29^ Regiment with dexamethasone or betamethasone was connected with an additional risk of cognitive abnormalities, whereas no danger of neurodevelopmental abnormalities was observed with hydrocortisone. This observation has yet to be confirmed in a randomized controlled trial. Meanwhile, the severity of BPD may also have an impact on the neurodevelopmental outcome, which is related to both glucocorticoid usage and BPD severity. In a single‐center retrospective cohort study of GA <30 weeks patients who survived to 36 weeks of PMA, Trixie et al. made the following assertions: NDI was statistically significant for all BPD severities compared with babies without BPD; among all infants with BPD, the risk of NDI increased 5‐fold between 2 and 5 years of age; the strength of the association between NDI and BPD severity did not change over time.^23^

Second, postnatal systemic steroid use increases the severity of retinopathy of prematurity (ROP). ROP is a leading reason of childhood blindness in developed countries, and it is now accepted that oxygen supply and prematurity are the main risk factors for ROP.^30^ Other identified risk factors include MV, BPD, intraventricular hemorrhage (IVH), necrotizing tiny bowel colitis (NSC), blood transfusions, and sepsis. Postnatal systemic steroids are known to increase the risk of ROP. However, it is not known whether the accumulated dose and type of postnatal systemic steroids are connected with the development and severity of ROP.^30^ Katsuo included 75 newborns born at <28 postmenstrual weeks (PMA) in a multifactorial logistic regression analysis showing that gross systemic steroid dose was the single risk factor for severe ROP requiring photocoagulation (PC) and that a cutoff value of 8.95 mg/kg for postnatally administered systemic steroids could be used as a valuable marker for predicting severe ROP requiring PC.^31^

Third, postnatal glucocorticoid use affects renal function and blood pressure in very low birth weight (VLBW) neonates. In a retrospective cohort study, Christiane et al. examined medical records of infants born from January 2015 to December 2019 (≤35 weeks GA) to examine the cumulative dose and duration of glucocorticoids on blood pressure and renal function in VLBW infants. They discovered that when cumulative steroid dose rose, it caused systolic blood pressure and significantly increased creatinine clearance in newborns being discharged from the hospital. It suggests that the duration of postnatal steroid use and accumulated dose are connected with increased systolic blood pressure. Although this effect may disappear after discontinuation of treatment, so we should pay attention to prevent long‐term cardiovascular and renal diseases. Beyond that, in susceptible infants, increases in creatinine clearance and GFR in infants exposed to postnatal steroids have the possibility to cause short‐term improvements in renal function. This effect can mask the fact that renal injury is occurring. Animal studies have shown that exposure to high doses of glucocorticoids leads to renal cyst formation, renal unit loss, and renal morphogenesis.^32^ We should be more cautious when using postnatal cortisol in these extremely fragile newborns since its effects on renal function are currently underappreciated.

Finally, postnatal use of cortisol may result in metabolic disturbances. It has been demonstrated in animal experiments that postnatal administration of low doses of dexamethasone to neonatal rats results in growth retardation and impaired thermogenesis, which causes cold intolerance in neonatal pups. The cause of these impacts could be related to interference with brown adipocyte differentiation.^33^ In addition, by recording daily temperature changes in infants on glucocorticoids, postnatal systemic corticosteroid use was connected with more significant temperature fluctuations, suggesting that glucocorticoids may impair neonatal thermoregulation.

### 4.4 Different SG regimens

Because hydrocortisone's ability to minimize the occurrence of BPD is unknown and dexamethasone's short‐term and long‐term effects remain a concern, there has recently been a series of clinical searches for alternative components and SG regimens. Trials have been conducted with betamethasone, prednisolone, or methylprednisolone as alternatives to dexamethasone and hydrocortisone. Unfortunately, the quality of the data is too low to indicate their connection to a decline in mortality and the incidence rate of BPD. As a result, the Canadian Pediatric Society's recent position statement does not suggest using these agents.

Glucocorticoid regimens have been studied, including the timing of treatment initiation, cumulative dose effect, pulse dosing, and individualized regimens. First, regarding the timing of treatment initiation, Onland et al. included 16 tests, five of which included 797 newborns, examining early (<7 days) versus moderately early (8–14 days) or delayed (>3 weeks) initiation of dexamethasone therapy.^7^ Another study found no variation in the combined outcomes of death at PMA 36 weeks or occurrence of BPD, PMA at 28 days, and death at PMA 36 weeks between the allocation groups. For the incidence of BPD at 28 days PMA and 36 weeks PMA, newborns with delayed start (>21 days PMA) were more numerous than those with a moderately early start (8–21 days PMA).^8^ There are no neurodevelopmental findings for infants whose medications were started at different times.

Second, for the effect of cumulative dose, a total of 306 participants were enrolled in eight cumulative dose studies. According to cumulative dose, these studies were divided into three categories: low cumulative dose (2 mg/kg), medium cumulative dose (between 2 and 4 mg/kg), and high cumulative dose (>4 mg/kg). No discrepancy existed between the high and low accumulated doses concerning abnormal neurodevelopmental outcomes assessed in BPD survivors, composite outcome, death, or BPD. On the contrary, there was no proof that a high cumulative dose increased the incidence of CP when compared to a low‐dose strategy.

The danger of CP was more severe with the highest accumulated dose than with the medium accumulated dose. In subgroup analyses comparing high‐dose dexamethasone with the medium accumulated dose regimen, there was more evidence of an elevated risk of death or CP with the highest cumulative dose. There was no difference in the primary outcome between the moderate and low cumulative doses.

Third, to the pulsed‐dose studies, which essentially refer to the administration of 0.5 mg/kg/day of dexamethasone for 3 days followed by 7 days without corticosteroid therapy, two studies comparing pulsed therapy with a continuous regimen of reduced doses showed an increased risk of death or BPD when using pulsed therapy with no difference in primary outcome or long‐term neurodevelopmental outcomes.^34^

Finally, as for the research on individualized regimens, it has been demonstrated that BPD has different pathological types and that patients differ in terms of gestational age, birth weight, prenatal steroid exposure, and mechanical or non‐mechanical ventilation, which means that the characteristics of BPD vary from individual to individual.^35^ In response to this characteristic, individualized drug administration has been proposed in recent years. However, a systematic review and meta‐analysis did not show significant differences between different dexamethasone regimens. The duration of MV was the only referenced short‐term outcome that differed, and it was significantly reduced by personalized and adjusted dosage regimens.

### 4.5 Applications of systemic hydrocortisone

Hydrocortisone is a synthetic cortisol analog with glucocorticoid activity but exhibits saline corticosteroid activity at physiologic doses. The current clinical practice is early use with low doses of hydrocortisone, which at low doses can circumvent some of the short‐term and long‐term adverse impacts of dexamethasone. However, the efficacy and safety of hydrocortisone have yet to be fully established.

In a systematic review and meta‐analysis, high COE showed that hydrocortisone administration in the first week was relevant to a reduced risk of BPD or mortality in newborns born at less than 32 weeks, whereas high COE showed that the early use of low doses of hydrocortisone had something to do with a reduced danger of BPD or mortality at 36 weeks. The moderate COE suggests that the late use of hydrocortisone helps reduce the risk of BPD or mortality at 36 weeks of PMA; the benefit of late hydrocortisone use is not supported by randomized controlled trials.^6^ The efficacy and safety of hydrocortisone were evaluated by Onland et al., who divided infants into three groups based on start times, cumulative doses, and administration times. Although the obvious distinction in the frequency of BPD and the distribution of BPD severity between the hydrocortisone group and the control group were unfounded, the data indicated that the extubation rate was more significant on day 3 in the hydrocortisone group than in the placebo group.^7^ The scientists also discovered that babies receiving hydrocortisone treatment had a higher risk of having severe IVH and necrotizing enterocolitis (NEC), which may be related to higher cortisol levels. More randomized controlled trials are needed to help identify these highly susceptible babies so that clinical decisions about the advantages and disadvantages of tailoring hydrocortisone treatment can be made. In these highly susceptible newborns, the potentially fatal negative consequences linked to hydrocortisone treatment are of greater concern than the advantages of improved BPD‐free survival.^20^ It is unknown whether using hydrocortisone after the second week of life improves survival in the absence of BPD and the absence of adverse neurodevelopmental effects. The survival without moderate or severe BPD at 36 weeks of PMA with hydrocortisone was not statistically different from that of the placebo group, according to a study of 800 babies under 30 weeks' GA who were intubated for at least seven days at 14–28 days (396 of whom received hydrocortisone).^25^ That hydrocortisone cure did not alter the severity of BPD assessed then, which could be related to identified inflammatory damage at late treatment.

Hydrocortisone now appears promising for improving short‐term prognosis without affecting long‐term neurodevelopment, which may be related to its mechanism of action. In animal studies, hydrocortisone did not show brain growth‐limiting impacts or apoptotic impacts on the hippocampus like dexamethasone, so hydrocortisone could be able to replace dexamethasone in terms of long‐term neurodevelopmental outcomes.^36^ However, the long‐term safety of hydrocortisone has not been demonstrated. Few adverse effects have been reported with hydrocortisone with short‐term adverse effects being hyperglycemia and hypertension. Concerns about long‐term neurodevelopmental outcomes, many trials have found no proof of long‐term neurologic damage through follow‐up visits of neurocognitive outcomes at 2 and 5 years of age. More extended follow‐up periods are needed to determine the long‐term safety of hydrocortisone. Table 1 summarizes the current clinical features regarding the use of SG.

**TABLE 1:** Pathologic and clinical features of different systemic glucocorticoids.

Type of treatment	Evidence	Clinical features	Comment
Early systemic glucocorticoids (dexamethasone or hydrocortisone)	Large meta‐analyses^26^	Almost no effect on death/BPD at 28 days PNA and 36 weeks PMA ↑Cerebral palsy ↑Gastrointestinal perforation ↑Combined mortality ↓Failure to extubate ↑Hyperglycemia ↑Hypertension	Not recommended
Early systemic hydrocortisone	Systematic review and meta‐analysis^7^ ^,^ ^20^	↓BPD/mortality ↑Extubation rate ↑Severe IVH and NEC ↑Hyperglycemia ↑Hypertension Doubt on neurodevelopment	Not recommended, needing further studies
Late systemic glucocorticoids (dexamethasone or hydrocortisone)	Large meta‐analyses^27^	↓BPD at 28 days PNA and 36 weeks PMA ↓Mortality ↑Extubation rate Almost no effect on cerebral palsy and the combined outcome ↓IVH ↑Hyperglycemia ↑Hypertension	To evaluate the benefit and the risk
Late systemic hydrocortisone	Meta‐analysis and RCT^6^, ^7^	↓BPD or mortality but RCT does not support.	Not recommended

Abbreviations: BPD, bronchopulmonary dysplasia; IVH, intraventricular hemorrhage; NEC, necrotizing enterocolitis; PMA, postmenstrual age; PNA, postnatal age; RCT, randomized controlled trial.

## 5 CONCLUSION

Postnatal SG, particularly dexamethasone, may reduce the incidence and severity of BPD. However, the early use of dexamethasone often leads to long‐term abnormal neurodevelopmental abnormalities, and its routine use is not recommended. The early use of hydrocortisone may be associated with a low risk of BPD or mortality, and no obvious neurodevelopmental abnormalities have been observed in children aged 2–5 years. Late SG reduces mortality or BPD at PMA of 36 weeks without a significant increase in CP. However, a longer follow‐up is still needed to ensure long‐term safety. Current research on alternative regimens to glucocorticoids, such as alternative formulations, lower cumulative doses, pulsed dosing, and individualized dosing, has not yet produced high‐quality evidence of their efficacy and safety, and further clinical trials are necessary. On account of the available evidence, careful clinical judgment is needed to weigh the benefits and risks of SG in the management of BPD.

## AUTHOR CONTRIBUTIONS

Xinyi Wang collected and sorted out the literature and wrote the first manuscript draft. Yuan Shi was the guarantor and revised it critically for important intellectual content.

## CONFLICT OF INTEREST STATEMENT

Yuan Shi is the Deputy Editor‐in‐Chief of *Pediatric Discovery*. He was excluded from all editorial decision‐making related to the acceptance of this article for publication. The remaining authors declare no conflict of interest.

## ETHICS STATEMENT

Not applicable.

## Data Availability

Data sharing is not applicable to this article as no datasets were generated or analyzed during the current study.
